# Nanoscale Ring-Shaped Conduction Channels with Memristive Behavior in BiFeO_3_ Nanodots

**DOI:** 10.3390/nano8121031

**Published:** 2018-12-11

**Authors:** Zhongwen Li, Zhen Fan, Guofu Zhou

**Affiliations:** 1 Institute for Advanced Materials, South China Academy of Advanced Optoelectronics, South China Normal University, Guangzhou 510006, China; chineseli_1982@163.com; 2 Guangdong Provincial Key Laboratory of Optical Information Materials and Technology, South China Academy of Advanced Optoelectronics, South China Normal University, Guangzhou 510006, China; guofu.zhou@m.scnu.edu.cn; 3 Faculty of Mathematics and Physics, Huaiyin Institute of Technology, Huai’an 223003, China; 4 National Center for International Research on Green Optoelectronics, South China Normal University, Guangzhou 510006, China; 5 Shenzhen Guohua Optoelectronics Tech. Co. Ltd., Shenzhen 518110, China; 6 Academy of Shenzhen Guohua Optoelectronics, Shenzhen 518110, China

**Keywords:** ring-shaped conduction channels, memristors, nanodots, BiFeO_3_

## Abstract

Nanoscale ring-shaped conduction channels with memristive behavior have been observed in the BiFeO_3_ (BFO) nanodots prepared by the ion beam etching. At the hillside of each individual nanodot, a ring-shaped conduction channel is formed. Furthermore, the conduction channels exhibit memristive behavior, i.e., their resistances can be continuously tuned by the applied voltages. More specifically, a positive (negative) applied voltage reduces (increases) the resistance, and the resistance continuously varies as the repetition number of voltage scan increases. It is proposed that the surface defects distributed at the hillsides of nanodots may lower the Schottky barriers at the Pt tip/BFO interfaces, thus leading to the formation of ring-shaped conduction channels. The surface defects are formed due to the etching and they may be temporarily stabilized by the topological domain structures of BFO nanodots. In addition, the electron trapping/detrapping at the surface defects may be responsible for the memristive behavior, which is supported by the surface potential measurements. These nanoscale ring-shaped conduction channels with memristive behavior may have potential applications in high-density, low-power memory devices.

## 1. Introduction

Nanoscale conduction channels with ring-like shapes are of particular interest in nanoscience owing to their broad application prospects. For example, they may be used to carry persistent currents [1,2]. In addition, when the ring-shaped conduction channels are made of magnetic materials, they may be used for constructing magnetic tunnel junctions with reduced power consumption and enhanced thermal stability [3,4]. Another appealing example is the metal oxides-based ring-shaped conduction channel, which shows excellent gas sensing properties due to a large surface-area-to-volume ratio [5,6]. Clearly, the ring-shaped conduction channels possess a vast number of functionalities, but their memristive properties seem to remain almost unexplored.

The memristive behavior manifests itself as a continuous change in resistance with the amount of charge flown across the device (i.e., the memristor). Therefore, multiple resistance states could be achieved by varying the amplitude and duration of applied voltage in a memristor, which is different from the conventional resistive switching between only two bi-stable resistance states. Nanoscale memristors can be used as synapses in brain-mimicking neuromorphic computational architectures. While most previous memristors were based on the planar capacitor structures [7,8,9,10,11,12,13,14], those based on new structures may yield opportunities to create new functionalities or improve the existing performance. For example, the memristors based on the above-mentioned ring-shaped conduction channels may consume less power compared with those based on the planar capacitor structures because of the smaller effective area for the charge flow (assuming that the power density is uniform along the in-plane direction). Thus, we aim to achieve such kind of nanoscale conduction channels possessing both ring-like shapes and memristive properties, which may have potential applications in high-density, low-power memory devices.

In terms of the material selection, we use BiFeO_3_ (BFO), which is a prototype multiferroic material. It exhibits a large polarization of ~60 µC/cm^2^ along the [001] direction [15,16], weak magnetism [17,18], and a rich palette of crystalline phases [19,20,21] and domain structures [22,23,24]. In recent years many intriguing properties have been discovered in BFO, particularly the domain wall conduction [25,26]. We previously found that BFO nanodots could exhibit unique topological domain structures, and thus novel conduction properties in association with the domain structures may be expected [27]. Herein, we report the observation of ring-shaped conduction channels with memristive behavior in BFO nanodots. Each ring-shaped conduction channel is formed at the hillside of each individual nanodot, and the resistance of the conduction channel can be continuously tuned by the applied voltage. The origins of the formation of ring-shaped conduction channels and their memristor behavior will be analyzed in relation to the surface defects, which are formed due to the etching and temporarily stabilized by the topological domain structures of BFO nanodots.

## 2. Materials and Methods

### 2.1. Materials Preparation

Epitaxial BFO films (~100 nm in thickness) with ~50 nm SrRuO_3_ (SRO) buffer layers were grown on (001)-oriented SrTiO_3_ (STO) substrates by using pulsed laser deposition (PLD). During the deposition of BFO films, the temperature was kept at 600 °C, and the oxygen pressure was fixed at 2–3 Pa. Then, a ~300-nm-thick anodic aluminum oxide (AAO) membrane mask was transferred onto the BFO film, followed by the Ar+ ion beam etching. Finally, the AAO mask was removed mechanically and the BFO nanodot arrays were obtained. The resultant BFO nanodots were free from the contaminations from residual AAO mask (see Figure S1 for evidence). 

### 2.2. Characterization

Nanoscale electrical characterizations based on scanning probe microscopy (SPM), including conductive atomic force microscopy (C-AFM) and scanning Kelvin probe microscopy (SKPM), were performed using an integrated SPM system (Asylum Cypher, Oxford Instruments, Abingdon, UK) equipped with conductive Pt-coated silicon probes (EFM Arrow, Nanoworld, Neuchâtel, Switzerland). The C-AFM and SKPM were used to characterize the current and surface potential, respectively. In the C-AFM measurement, voltages of ±1 V were used for the current mapping while voltages up to ±10 V were applied for measuring *I-V* characteristics. In the SKPM measurement, the tip was lifted 50 nm above the sample surface and an AC drive amplitude of 1 V was used. All the SPM-based studies were conducted in the ambient circumstance. 

## 3. Results and Discussion

**Figure 1:**
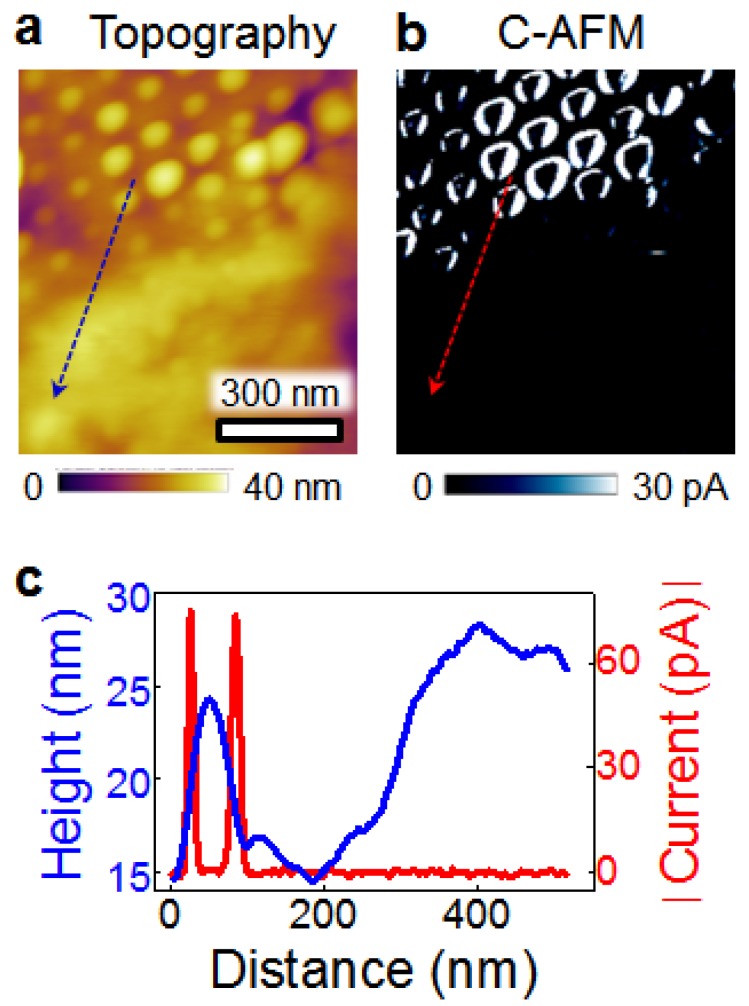
Comparison of topography and current response between BiFeO_3_ (BFO) nanodots and unetched film. (**a**) Topography and (**b**) current map (scanned with +1 V) of BFO nanodots and unetched film. In the current map, the nanodots show ring-shaped conduction channels (white color), while the unetched film shows negligible current (black color). (**c**) Height and current profiles along the section lines in a and b, respectively.

The structural characterizations of the BFO nanodots can be found in our previous work [27], while this work mainly deals with the electrical characterizations. The applied voltage is termed as positive if a positive bias is applied to the Pt tip, and the current flowing from the tip to the bottom electrode is defined as a positive current. Figure 1a presents the topography of a specific area containing both the BFO nanodots and the unetched BFO film. It is seen that the BFO nanodots have relatively uniform sizes, with an average height of ~25 nm and an average diameter of ~60 nm. The current map measured by a +1 V scan (Figure 1b) shows that there is no measurable current in the region of unetched film, whereas the ring-shaped conduction channels are distributed in the region of BFO nanodots. The conduction channels are at the hillsides of the nanodots, which exhibit large currents of ~80 pA (Figure 1c). However, the peaks of the nanodots are much less conductive, with currents of below ~1 pA (Figure 1c). The direct formation of ring-shaped conduction channels in the BFO nanodots circumvents the need for the fabrication of BFO nanorings, which simplifies the fabrication procedure and saves cost. We note that similar ring-shaped conduction channels were also observed in GeSi nanodots [28,29]. 

**Figure 2:**
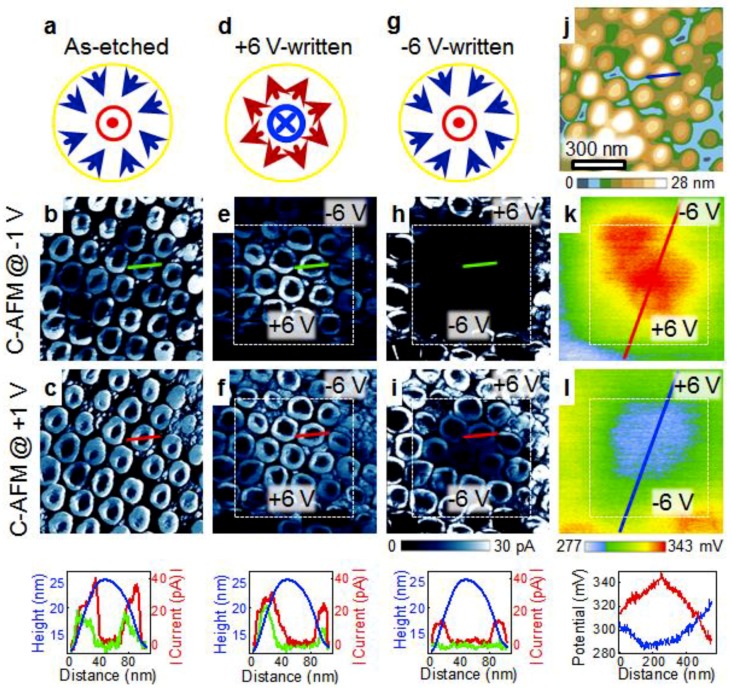
Domain structures, current maps and surface potential images before and after electrical writing. (**a**,**d**,**g**) Domain structures, and current maps scanned with (**b**,**e**,**h**) −1 V and (**c**,**f**,**i**) +1 V for the BFO nanodots: (**a**,**b**,**c**) in the as-etched state, (**d**,**e**,**f**) after writing with −6 V (outer)/+6 V (inner), and (**g**,**h**,**i**) after writing with +6 V (outer)/−6 V (inner). (**j**) Topography image of the BFO nanodots. Surface potential images taken after writing with (**k**) −6 V (outer)/+6 V (inner) and (**l**) +6 V (outer)/−6 V (inner). Insets at the bottom show the height, current and surface potential profiles along the section lines.

Turning to the origins of the formation of ring-shaped conduction channels in our BFO nanodots, we mainly consider two factors as follows:

(1) From a BFO film to the nanodots, the surface of the BFO film is subject to etching. This creates abundant surface defects, e.g., oxygen vacancies [30], which can lower the Schottky barrier at the Pt tip/BFO interface. We argue that the Pt/BFO Schottky barrier is mainly controlling the current flow in the Pt/BFO/SRO nanodevices for the following reasons. First, the BFO/SRO barrier is ruled out as the current-limiting barrier; otherwise, the BFO nanodots and the unetched film would have similar conductance, which is in contradiction with our observation (Figure 1b). Second, the bulk resistance of the BFO nanodot is also not the main factor limiting the current; otherwise, the currents measured with the positive and negative applied voltages would be similar in magnitude. However, it is clearly seen from Figure 2b,c that the currents at +1 V are higher than those at −1 V. We therefore deduce that the Pt/BFO Schottky barrier is mainly controlling the current flow. The +1 V and −1 V correspond to the forward and reverse bias conditions, resulting in asymmetric conductance. In addition, because the hillsides of the nanodots are etched more severely than the peaks, a larger number of surface defects are thus distributed at the hillsides, making the Schottky barriers there much lower. This seems to be the main factor leading to the formation of ring-shaped conduction channels. 

(2) We speculate that the center-convergent-type topological domain structures of the as-etched BFO nanodots (Figure 2a and Figure S2) may temporarily stabilize the surface defects at the hillsides. More specifically, the negative polarization charges near the surfaces of hillsides may help to stabilize the positively charged oxygen vacancies, preventing them from diffusing away or interacting with the atmosphere [31,32]. This speculation is supported by the following experiment. A region of the film was fully etched without covering the AAO mask, and then the current map was measured. Interestingly, negligible currents are observed in this region (Figure S3), despite the fact that the amount of etching-induced surface defects is also large. To understand this, we checked the domain structures in the fully etched region. It is found that in this region almost all the domains are pointing upward (Figure S3). The positive polarization charges near the film surface may disfavor the positively charged oxygen vacancies. As a result, the oxygen vacancies may either diffuse away or be compensated by the trapped electrons from the atmosphere [33]. Therefore, the lowering of the Schottky barrier induced by the oxygen vacancies is ineffective in the fully etched film. The difference in conductance between the fully etched film and the nanodots thus suggests that center-convergent-type topological domain structures of the nanodots may temporarily stabilize the surface defects, contributing to the formation of ring-shaped conduction channels. In addition, the observation of negligible currents in the fully etched film indicates again that the thickness of the film (i.e., the bulk resistance of the film) is not a major factor in influencing the current. 

Then, we proceed to investigate the modulation of ring-shaped conduction channels by the applied voltages. The BFO nanodots were first electrically written with large applied voltages of ±6 V; the resultant domain structures and current maps are shown in Figure 2d–i. As can be seen from Figure 2d,g and Figure S2, the applied voltages of +6 V and −6 V can lead to the formation of center-divergent and center-convergent domains, respectively (see our previous work [27] for details). Under small reading voltages (both +1 V and −1 V), the ring-shaped conduction channels in the +6 V-written region are still observable, while those in the −6 V-written region become less conductive or even disappear (Figure 2e,f,h,i). Moreover, the surface potential images (Figure 2k,l) show that the +6 V-written region has a higher surface potential than the −6 V-written region. 

To interpret the above results, it is crucial to determine the electrical processes which occur during the ±6 V writing. It was suggested that high-voltage writing could induce not only the domain switching but also the charge injection/extraction [34,35] and the filaments’ formation/rupture [36,37]. The filamentary mechanism is not suitable for the observed resistive switching, because the conduction channels have a quite uniform distribution and no forming step is required to initiate the resistive switching. 

**Figure 3:**
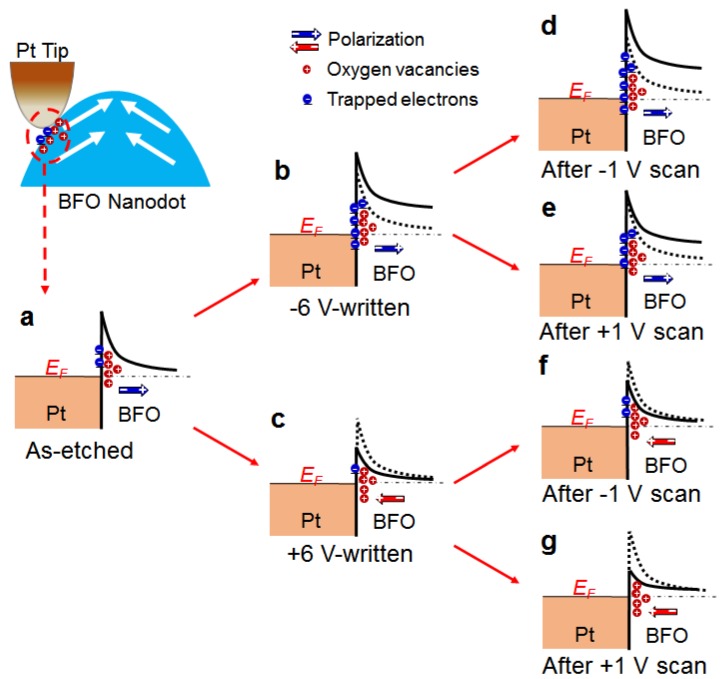
Schematic energy band diagrams in different states. Schematic energy band diagrams at the Pt tip/BFO interface of (**a**) as-etched, (**b**) −6 V-written, and (**c**) +6 V-written states. Inset on the top of Panel a indicates the actual interface between the Pt tip and the BFO nanodot. The Schottky barrier in the −6 V-written state further modified by the (**d**) −1 V and (**e**) +1 V scans, and that in the +6 V-written state further modified by the (**f**) −1 V and (**g**) +1 V scans.

We thus turn to the charge injection/extraction, an area-type resistive switching. More specifically, during the −6 V writing, abundant electrons may be injected from the Pt tip and subsequently trapped at the surface defects, which could compensate or even over-compensate for the positive charges of oxygen vacancies (Figure 3a,b). In contrast, during the +6 V writing, the reverse scenario, i.e., electron detrapping, may occur, making the oxygen vacancies uncompensated or less compensated for (Figure 3a,c). Because the surface potential is influenced by all the charged species near the surface, including the polarization, oxygen vacancies, and trapped electrons [38], it is deducible that the surface potential of the +6 V-written region is higher than that of the −6 V-written region, consistent with our observation (Figure 2k,l). In addition, a higher surface potential indicates a lower Schottky barrier for the electron transport [39], as schematically shown in Figure 3b,c. This therefore explains the observation that the BFO nanodots in the +6 V-written region exhibit larger currents than those in the −6 V-written region (Figure 2e,f,h,i). 

**Figure 4:**
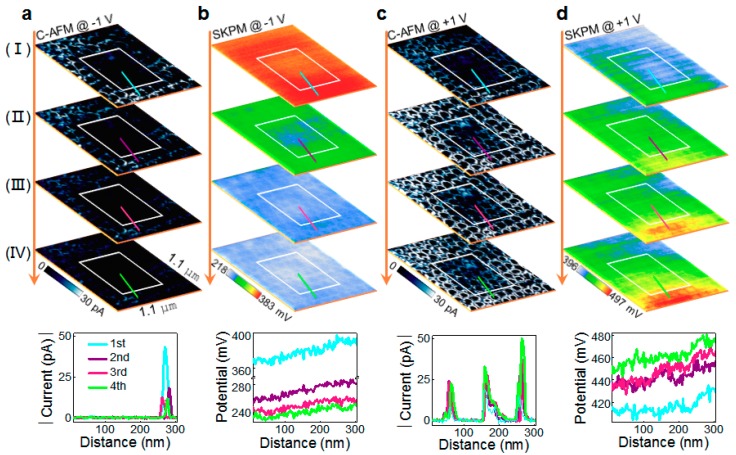
Current maps and surface potential images after multiple ±1 V scans. Current maps scanned with (**a**) −1 V and (**c**) +1 V four times (I–IV) in a region initially written with +6 V (outer)/−6 V (inner). Surface potential images taken right after the (**b**) −1 V and (**d**) +1 V scans (I–IV). Insets at the bottom show the current and surface potential profiles along the section lines.

Moreover, we also observe the memristive behavior of the ring-shaped conduction channels in our BFO nanodots. Multiple voltage scans with the small voltages of ±1 V were applied to the region which was initially written with +6 V(outer)/−6 V(inner), and for each voltage scan a current map was recorded. Figure 4a shows that as the number of −1 V scan increases, the conduction channels in the +6 V-written region become less conductive while the currents in the −6 V-written region remain almost zero. However, as the number of +1 V scan increases, the currents in the +6 V-written region become larger while the conduction channels start to emerge in the −6 V-written region (Figure 4c). These results demonstrate that the conduction channels exhibit the memristive behavior which can be operated under small applied voltages of ±1 V.

The memristive behavior may be related to electron trapping/detrapping, as supported by the surface potential measurements. As shown in Figure 4b, the surface potential becomes successively lower by increasing the number of −1 V scans. However, the converse change in surface potential is observed in the case of +1 V scan (Figure 4d). These results suggest that electron trapping (detrapping) may occur during each −1 V (+1 V) scan, thus lowering (enhancing) the surface potential. As a result, the Schottky barrier at the Pt tip/BFO interface may become higher (lower), reducing (increasing) the conductance of the conduction channel (see Figure 3d–g). Also note that the voltage scans with ±1 V can hardly induce the domain switching (see Figure S4 for evidence). Therefore, the electron trapping/detrapping, which may even occur under small applied voltages, appears to be the origin of the memristive behavior. 

**Figure 5:**
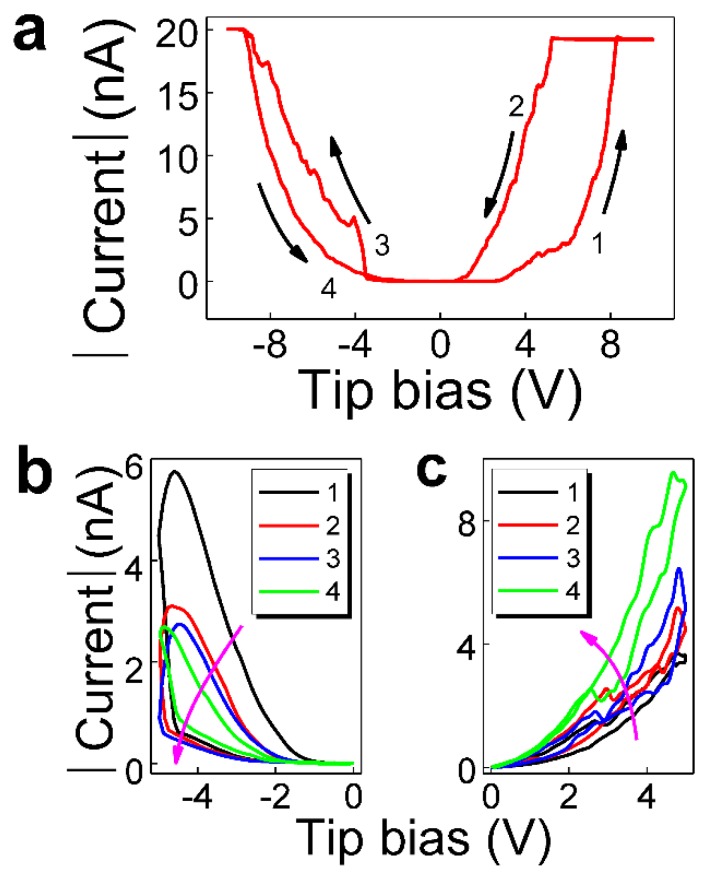
Memristive properties of individual conduction channels. Typical current–voltage (*I-V*) characteristics measured under (**a**) a bipolar voltage scan of 0 → +10 V → 0 → −10 V → 0, and unipolar voltage scans of (**b**) 0 → −5 V → 0 and (**c**) 0 → +5 V → 0.

Besides the current mapping, the current–voltage (*I-V*) measurements were also conducted to study the memristive properties of individual conduction channels. Figure 5a shows the hysteretic *I-V* characteristics measured by placing the tip at the hillside of a randomly-selected nanodot. As the voltage scans from 0 to +10 V, the conduction channel exhibits a transition from high resistance state (HRS) to low resistance state (LRS) at ~+8 V. The conduction channel remains in the LRS during the voltage scan of +10 V → −8 V. When the voltage exceeds ~−8 V, the conduction channel is turned to the HRS, and this HRS is maintained until the voltage goes back to 0. These features of *I-V* characteristics indicate that the bipolar-type resistive switching occurs in the conduction channels. 

We further measured the *I-V* characteristics by applying unipolar positive and negative voltages. It is seen from Figure 5b that the resistance continues to increase as the number of negative voltage scans (0 → −5 V → 0) increases. For example, the absolute value of current read at −2 V in the 1st cycle is 0.68 nA, and it decreases to 0.14, 0.13, and 0.07 nA in the 2nd, 3rd and 4th cycles, respectively. On the contrary, as shown in Figure 5c, the resistance continues to decrease as the number of positive voltage scans (0 → 5 V → 0) increases. For example, the absolute value of current read at 2 V in the 1st cycle is 0.41 nA, and it increases to 0.60, 0.68, and 1.55 nA in the 2nd, 3rd and 4th cycles, respectively. These results demonstrate that the individual conduction channels exhibit memristive behavior, consistent with the results of current mapping.

## 4. Conclusions

In summary, we have observed nanoscale ring-shaped conduction channels with memristive behavior in BFO nanodots prepared by ion beam etching. The conduction channels are located at the hillsides of the nanodots. Furthermore, the conduction channels exhibit memristive behavior, i.e., their resistances can be increased (decreased) successively by increasing the number of negative (positive) voltage scans. We propose that the surface defects distributed at the hillsides of nanodots may lower the Schottky barriers at the Pt tip/BFO interfaces, thus leading to formation of ring-shaped conduction channels. The surface defects are formed due to the etching and they may be temporarily stabilized by the topological domain structures of BFO nanodots. In addition, the memristive behavior may originate from the electron trapping/detrapping, which is supported by the surface potential measurements. These nanoscale ring-shaped conduction channels with memristive behavior may have potential applications in high-density, low-power memory devices.

## Supplementary Materials

The following are available online at http://www.mdpi.com/2079-4991/8/12/1031/s1. Figure S1: (a) Top-view SEM image, and (b) EDS spectra of BFO nanodots. Figure S2: PFM vertical phase images, lateral phase images, and corresponding domain structures of (a) as-etched and −6 V-written, and (b) +6 V-written BFO nanodots. Figure S3: (a) Topography and (b) current images of a specific area containing both BFO nanodots and fully-etched film. (c) Height and current profiles along the section lines in a and b, respectively. (d) PFM phase image taken in a fully-etched region with the middle region written by +6 V. Figure S4: PFM phase images (a) before and (b) after multiple ±1 V scans.
