# Supplementary material for: Nanoscale Ring-Shaped Conduction Channels with Memristive Behavior in BiFeO3 Nanodots
Source: Nanomaterials (Basel). 2018 Dec 11;8(12):1031. doi: 10.3390/nano8121031 (PMC6315444; doi:10.3390/nano8121031)
Supplement: Supplementary file 1 [file nanomaterials-08-01031-s001.pdf]

Supplementary Materials

Nanoscale Ring-shaped Conduction Channels with Memristive Behavior in $BiFeO_3$ Nanodots

Zhongwen Li [1,2,3], Zhen Fan [1,2*], Guofu Zhou [2,4,5,6]

[1] Institute for Advanced Materials, South China Academy of Advanced Optoelectronics, South China Normal University, Guangzhou 510006, China; chineseli_1982@163.com
[2] Guangdong Provincial Key Laboratory of Optical Information Materials and Technology, South China Academy of Advanced Optoelectronics, South China Normal University, Guangzhou 510006, China; fanzhen@m.scnu.edu.cn
[3] Faculty of Mathematics and Physics, Huaiyin Institute of Technology, Huai'an, 223003, China
[4] National Center for International Research on Green Optoelectronics, South China Normal University, Guangzhou 510006, China; guofu.zhou@m.scnu.edu.cn
[5] Shenzhen Guohua Optoelectronics Tech. Co. Ltd., Shenzhen 518110, China
[6] Academy of Shenzhen Guohua Optoelectronics, Shenzhen 518110, China
* Correspondence: fanzhen@m.scnu.edu.cn; Tel.: +86-15626158356 (Z.F.)

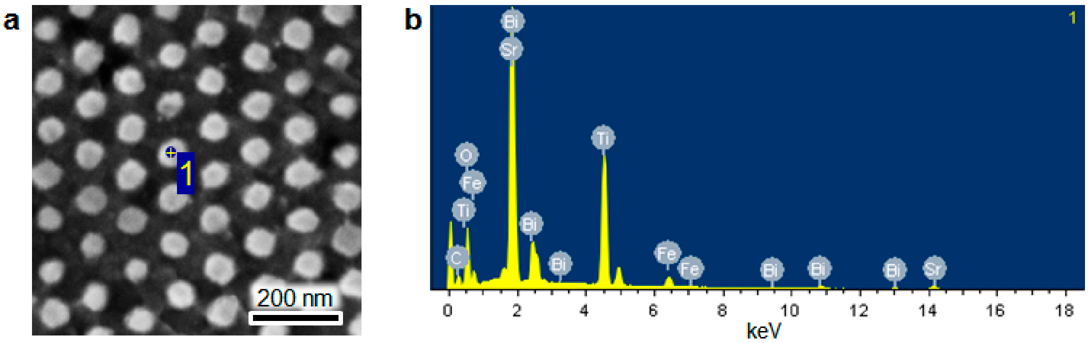

Figure S1. (**a**) Top-view scanning electron microscope (SEM) image of BFO nanodots. (**b**) Energy dispersive spectroscopy (EDS) spectra of BFO nanodots taken in the area marked in a. As can be seen, no signals from the element Al are detected, evidencing that there are no contaminations from the residual AAO mask.

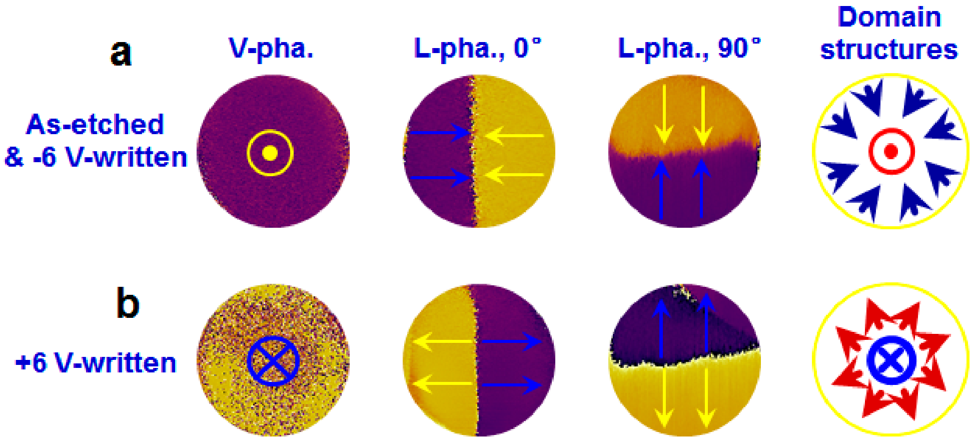

Figure S2. The piezoresponse force microscopy (PFM) vertical phase images, lateral phase images, and corresponding domain structures of (**a**) as-etched and -6 V-written, and (**b**) +6 V-written BFO nanodots. The domain structure in a is center-convergent-type, while that in b is center-divergent-type. The method to derive the domain structures was described in detail in our previous work [Li et al., Sci. Adv. 3, e1700919 (2017)].

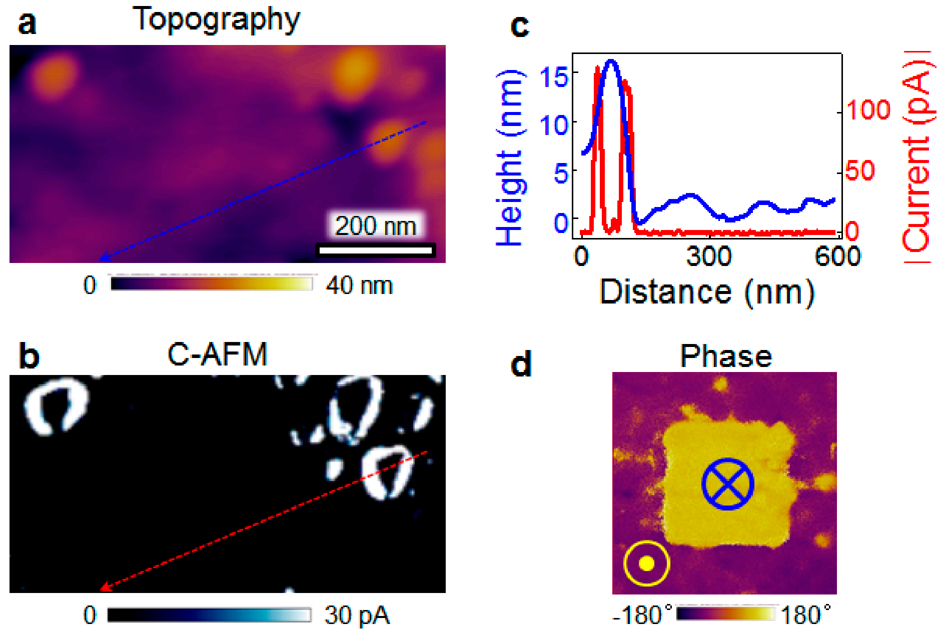

Figure S3. (**a**) Topography and (**b**) current images of a specific area containing both BFO nanodots and fully-etched film. (**c**) Height and current profiles along the section lines in **a** and **b**, respectively. (**d**) PFM phase image taken in a fully-etched region with the middle region written by +6 V. Because the fully-etched region and the +6 V-written region exhibit a phase difference of 180°, the domains in the fully-etched region are thus mostly oriented upward.

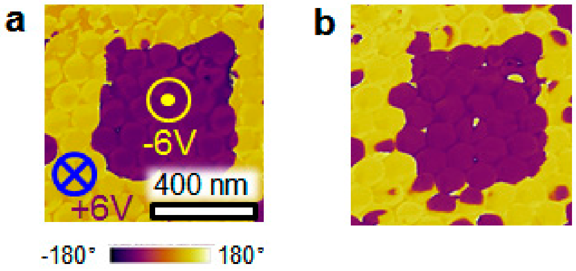

Figure S4. PFM phase images (**a**) before and (**b**) after multiple ±1 V scans. There are almost no changes in the phase contrast after the multiple ±1 V scans.
